# The public washroom - friend or foe? An observational study of washroom cleanliness combined with microbiological investigation of hand hygiene facilities

**DOI:** 10.1186/s13756-019-0500-z

**Published:** 2019-02-28

**Authors:** Lorna K. P. Suen, Gilman K. H. Siu, Yue Ping Guo, Simon K. W. Yeung, Kiki Y. K. Lo, Margaret O’Donoghue

**Affiliations:** 1 School of Nursing, The Hong Kong Polytechnic University, Hung Hom, Hong Kong, SAR China; 2 Department of Health Technology and Informatics, The Hong Kong Polytechnic University, Hung Hom, Hong Kong, SAR China

**Keywords:** Washroom, Environmental microbiology, MALDI-TOF MS, Hand hygiene, Antimicrobial, Bacteria, Microorganisms, Public health, Toilet, Hand drying

## Abstract

**Background:**

Many people use handwashing and hand-drying facilities in public washrooms under the impression that these amenities are hygienic. However, such facilities may be potential sites for the transmission of pathogenic bacteria. This study aimed to examine the hygiene facilities provided including handwashing and hand-drying facilities in public washrooms. Total bacterial counts and species identification were determined for hand-drying facilities. Antimicrobial susceptibilities were performed.

**Methods:**

The bacterial contamination levels of 55 public washrooms ranging in category from low class communities to high end establishments, were examined. The hygienic environment and facilities of the washrooms were analysed using an electronic checklist to facilitate immediate data entry. Pre-moistened sterile swabs were used to collect samples from areas around the outlet of paper towel dispensers, air outlet of air dryers, exit door handles and paper towels in the washrooms. Total bacterial counts were performed and isolates identified using matrix-assisted laser desorption ionisation time-of-flight mass spectrometry. Antimicrobial susceptibility was determined by disk diffusion.

**Results:**

The high and middle-income categories washrooms generally had cleaner facilities and environment followed by those in low categories. Fifty-two bacterial species were identified from the 55 investigated washrooms. Over 97% of the pathogenic *Staphylococcus spp.* tested were resistant to at least one first-line antimicrobial therapeutic agent, including penicillin, cefoxitin, erythromycin, co-trimoxazole, clindamycin and gentamicin, and 22.6% demonstrated co-resistance to at least three antimicrobial agents, with co-resistance to penicillin, erythromycin and clindamycin being the most common.

**Conclusion:**

Our findings suggest that hand-drying facilities in public washrooms can act as reservoirs of drug-resistant bacteria. The importance of frequent cleaning and maintenance of public washrooms to promote safe hand hygiene practices for the public are emphasised.

**Electronic supplementary material:**

The online version of this article (10.1186/s13756-019-0500-z) contains supplementary material, which is available to authorized users.

## Introduction

Given their warm and humid environment, washrooms provide an ideal setting for the survival of microorganisms. Many pathogens, including *Shigella spp.*, *Escherichia coli*, *Pseudomonas aeruginosa*, *Acinetobacter baumanii, Staphylococcus aureus* and norovirus can survive on environmental surfaces for weeks or months [1–3]. Contaminated environments may also serve as vehicles for the acquisition and spread of methicillin-resistant *S. aureus* (MRSA) to the nose, eyes or mouth of washroom users via indirect contact [4, 5]. Contaminated areas not only include toilet facilities and their immediate environment [6, 7] but also hand-drying facilities. Contamination of paper towels and their dispensers has been demonstrated [8]. Aerosol generated from warm-air hand dryers may transmit pathogenic bacteria onto the hands and body of users [9]. Washroom users avail of the handwashing and hand-drying facilities provided under the impression that these amenities are hygienic. However, such facilities may be potential sites for the transmission of pathogenic bacteria. Little attention has been paid to the potential risks of re-contamination of hands from contaminated washroom facilities and surfaces. This study aimed to examine the cleanliness of the washroom environment and in particular, the hand-drying facilities of public washrooms. Given that countless people use public washrooms on a daily basis, the findings of this study may have significant public health implications.

## Materials and methods

### Study design

A cross-sectional study to determine cleanliness of the public washroom environment and facilities using observational data and microbiological analyses.

### Setting and procedures

This study was conducted between April and August 2017 to examine overall cleanliness and bacterial contamination levels of 55 public washrooms in Hong Kong via convenience sampling. The cleanliness of the environment and facilities of public washrooms was determined using an electronic checklist to facilitate on-the-spot data entry. To ensure an objective evaluation of the cleanliness of the washroom environment, an interrater reliability of over 95% among the research personnel conducting the assessments was ensured. The checklist consisted of three parts: Part 1 included questions on the overall environment of the washroom (including temperature, humidity and cleanliness). Part 2 included observations on the handwashing facilities provided; Part 3 focused on hand-drying facilities. To increase the generalisability of the findings, washrooms from different categories, ranging from high end (five-star hotels or restaurants), to middle (public libraries, shopping malls, sports centres, tourist spots and hospitals) and low categories (public housing estates and food markets), were evaluated.

Swabs pre-moistened in sterile normal saline were used to collect samples from areas around the outlet of paper towel dispensers, air outlet of air dryers, internal surface of exit door handles and paper towels in the washrooms. The swab was applied to the test area using a standardised zig-zag pattern of movement. The dimensions of the sampling area were recorded for subsequent calculation of the colony forming units (CFU) per cm^2^. Samples that could not be cultured immediately were stored at 4 °C and processed within 3 h of sampling [10]. To determine presence if any, of bacterial contamination on unused paper towels, the first two sheets from the dispenser were discarded and the next five sheets were collected aseptically into a sterile stomacher bag and transported immediately to the microbiology laboratory for further processing.

### Microbiological analyses

#### Total bacterial counts

Each swab was transferred to 1 mL of sterile Stuart’s solution and vortexed for 20 s. 0.1 mL of sample was aseptically transferred to Tryptone Soy Agar (TSA, Oxoid, UK) plates and spread evenly using a sterile L-shaped disposable spreader. All samples were cultured in duplicate and incubated aerobically at 37 °C for 48 h. Total bacterial counts were enumerated using an automated plate reader (BIOMIC V3 plate reader, BIOMIC, USA). CFUs per cm^2^ or per item tested were determined.

#### Bacterial identification

Individual colonies appearing to have different morphologies on the TSA were selected for further investigation (at least three distinct colonies per sample were selected). Rapid identification of isolates using matrix-assisted laser desorption ionisation time-of-flight mass spectrometry (MALDI-TOF MS) was performed. A tiny portion of an isolated colony was inoculated directly onto one spot of an MSP96 plate using a sterilised toothpick. On-plate protein extraction was performed by adding 70% formic acid (1 μl). *alpha*-Cyano-4-hydroxycinnamic acid (1 μl) matrix solution was then coated onto each target spot [7]. The target plate was analyzed using a Bruker Microflex LT system and MALDI Biotyper Compass software with the V5.0.0.0 spectra library (5989 spectra). Results were interpreted using a scoring process as recommended by the manufacturer. Scores of ≥2.0 implied high confidence of species identification, whereas scores between 1.70 and 1.99 were considered as intermediate-confidence for identification at the genus level. Scores of < 1.69 were considered unacceptable for identification.

#### Antimicrobial susceptibility testing

Disk diffusion testing was performed on all *S. aureus*, *S. saprophyticus,* and *S. epidermidis* strains following Clinical and Laboratory Standards Institute guidelines M100-S28 for antimicrobial susceptibility testing [11]. *S. saprophyticus* was selected because of its ability to cause urinary tract infection; and *S. epidermidis* because it is the most common coagulase negative *Staphylococcus.* The following antibiotics were tested: penicillin (10 units), Cefoxitin (30 μg; to test for methicillin resistance), erythromycin (15 μg), co-trimoxazole (1.3/23.8 μg), clindamycin (2 μg) and gentamicin (10 μg) [11].

### Data analyses

Descriptive statistics were used to analyse the checklist data collected on the cleanliness of the washroom environment, handwashing and hand-drying facilities and microbiological sampling results. Chi-square analyses were used to identify the association of specific variables (including washroom categories and gender of washrooms) with the washroom environment and cleanliness. Between-group comparisons using Mann–Whitney *U* test were conducted to determine significant differences in the total bacterial count of hand-drying facilities and the specific variables mentioned above. Analyses were conducted using SPSS version 25.0. Statistical significance was considered at *p* < 0.05.

## Results

### Washroom environment and facilities

A total of 55 public washrooms with equal gender distribution were examined. The average washroom temperature was 27.5 °C (± 1.9) and 62.4% humidity (±10.8%). The majority of the washrooms included a door entrance (*n* = 43, 78.2%). All the washroom toilets were supplied with tissue rolls, and 20% (*n* = 11) provided spare rolls. Nearly 90% of the rubbish bins were improperly closed, with almost 20% of the bins placed right below the hand dryers. Sanitary bins in the female washrooms were frequently uncovered (46.4%), with female napkins sometimes found overflowing or outside the bin. Over 85% of washrooms provide no handwashing signage/reminder. More than 50% of the washrooms lacked shelves or areas for placing personal belongings to facilitate handwashing. Although hand soaps or detergents were frequently supplied for users, the dispensers for hand sanitizers and paper towels were occasionally invisible to users (20%) and were oftentimes incorrectly positioned. Hands-free, motion sensor faucets were present in 89.0% of washrooms. However, only a small number of paper towel dispensers were autonomically-controlled (5%), and users frequently needed to manually obtain the paper towels from the dispensers either directly or by use of screw- or lever-controlled device. Warm-air hand-dryers, most of which were automatically controlled (83.3%), were more frequently provided (76.4%) than jet-air hand-dryers (10.9%) (Additional file 1: Table S1).

When compared with low-category washrooms, those in high and/or middle categories were significantly more likely to be supplied with toilet seat disinfectant (*p* < 0.05), have relatively cleaner environmental appearance in the toilet/urinal area (*p* < 0.05), floor areas (*p* < 0.001), walls (*p* < 0.01), and sinks (*p* < 0.05). The majority of the washrooms (76.4%) did not display log books to indicate cleaning schedules. Notably, female washrooms exhibited better overall cleanliness than male washrooms (Additional file 2: Table S2). No significant differences were noted in the total bacterial count of hand-drying facilities and specific variables (washroom category, gender).

### Results of microbiological testing

A considerable number of bacteria were isolated from the paper towel dispensers and hand dryers tested. The highest CFU/cm^2^ was found on internal door handles (1.48 × 10^2^), followed by jet air dryer (1.42 × 10^2^), warm air hand dryer (1.38 × 10^2^), paper towel (1.12 × 10^2^) and paper towel dispenser (0.9 × 10^2^). Overall, we identified 52 species from the 220 washroom samples collected. Potentially pathogenic gram negative rods such as *E. coli*, *Proteus mirabilis, Moraxella spp.,* and the gram-positive cocci, *S. aureus* and *S. saprophyticus* were isolated from the outlets of paper towel dispensers, hand dryers and/or door handles of a number of washrooms (Additional file 3: Table S3).

Antimicrobial susceptibility testing showed that over 87.1% (27 out of 31 samples) of the *Staphylococcal spp.* tested were resistant to at least one first-line antimicrobial therapeutic agent such as penicillin, cefoxitin, erythromycin, co-trimoxazole, clindamycin or gentamicin. 23%7/31) of samples exhibited co-resistance to at least three antimicrobial agents, with co-resistance to penicillin, erythromycin and clindamycin being the most common combination. The samples tested were obtained from paper towel dispensers, warm-air or jet-air hand-dryers or internal door handles from different categories (high, middle or low) of washrooms. No MRSA was detected but one methicillin resistant strain of *S. epidermidis* (MRSE) and *S. saprophyticus* were detected from a low category- (paper towel dispenser) and a middle category- (warm air dryer) washroom respectively. Both strains were additionally resistant to erythromycin and clindamycin. (Table 1).

**Table 1:** Antibiotic Resistance of *Staphylococcal spp* tested

Staphylococcal *spp.*	Total number of isolates	% Resistance (Number of isolates)						Resistance to ≥1 antibiotic(s)	Resistance to ≥3 antibiotics
		Penicillin	Cefoxitin	Erythromycin	Co-trimoxaxole	Clindamycin	Gentamicin		
*Staphylococcus aureus*	3	66.7% (2)	0% (0)	33.3% (1)	0% (0)	0% (0)	33.3% (1)	66.7% (2)	33.3% (1)
*Staphylococcus epidermidis*	24	70.8% (17)	4.2% (1)	62.5% (15)	0% (0)	12.5% (3)	0% (0)	91.7% (22)	16.7% (4)
*Staphylococcus saprophyticus*	4	50.0% (2)	25.0% (1)	75.0% (3)	0% (0)	75% (3)	0% (0)	75.0% (3)	50.0% (2)

## Discussion

The results of our study suggest that adequate hand hygiene may not always be achievable when using public washrooms. As might be expected, the higher end category of washrooms generally displayed a cleaner environment followed by those in the middle and low categories. The environmental surfaces of washrooms, especially where water droplets may collect, should be frequently cleaned. However the absence of log books in the majority of washrooms suggests that there might have no monitoring system to ensure regular cleaning of washrooms. It was interesting to note that all three *S. aureus* strains isolated were recovered from the internal door handles of males washrooms. It is possible that these strains were transferred from the hands of male washroom users due to failure to hand wash or inadequate technique. Gender is a highly significant predictor of hand washing behaviour [12]. In line with predictions, significantly more females than males reported that they perform hand washing with soap and water rather than water alone in all critical moments. In a study on determinants of hand-washing with soap and cleaning of household surfaces, Aunger et al. [13] reported that male respondents in their study may deter from performing hand hygiene behaviours if they are in a hurry, if no one else is in the washroom at the time, or when they have only urinated. Further studies are needed to understand the handwashing practices of both sexes in the community. Although studies have reported that both genders fall short on *Centers for Disease Control* and Prevention recommended hand washing durations (i.e. scrubbing for 20s before rinsing), women wash their hands significantly more often, use soap more often and wash their hands for longer than men (mean of 6.27 s for handwashing duration for males versus 7.07 s for females) [14]. That females exhibit better hand hygiene practices is further supported by our finding that female washrooms exhibited an overall cleaner condition than male washrooms.

Over 85% of the washrooms provided no handwashing signage/reminders for hand washing. The importance of using a positive tone rather than a fear-based one in visual prompts to remind washroom patrons to perform hand hygiene has been demonstrated. Fear-based messages (such as signage with flu warning) may discourage hand washing in public restrooms, as a fear-based message may provoke fear and trigger negative emotions in patrons that make the desired behavior less likely to change [15]. Therefore, the choice of words is critical to achieve the goal of hand hygiene. Special attention should be given to ensuring the prompt for encouraging hand hygiene is presented in a positive and supportive style.

Substantial numbers of bacteria were present on the paper towel dispensers, hand dryers and door handles tested. Such microbes could be easily transmitted between individuals by touching hand-drying facilities or the surrounding environment. Previous studies have shown that skin-associated bacteria are generally resilient and can survive for periods up to several years [16]. The majority of bacteria identified in this study are considered as part of the normal human flora and do not commonly cause disease in healthy individuals. For instances, *Brevibacterium spp.*, *Rothia spp.*, *Kocuria spp. Tsukamurella spp.,* and most of the *staphylococcus spp.* are skin flora of human [17], whereas *Corynebacterium spp., Neisseria spp. and Moraxella spp.* are known to colonize the oral cavity in healthy people [18]. Some organisms are ubiquitously present in the environment, such as *Bacillus spp.* and *Pseudomonas spp.* [19]. However, some species are known to be pathogenic to humans. *E. coli*, *Proteus mirabilis,* and *S. saprophyticus* are common urinary tract pathogens [20, 21]. *S. aureus* is the most frequent cause of community-associated skin and soft tissue infections [22].

Compelling evidence has shown that public restroom environments are frequently contaminated and show potential transmission of bacteria or viruses, including antibiotic-resistant bacteria [6, 7, 23]. A UK study [7] revealed that the drug resistance rate among *Staphylococcus spp.* isolated from public restrooms was 37.8%. In this study, about 67.7% (21/31) of *Staphylococcal* isolates were resistant to penicillin. However, it was surprising to note that over 20% of the isolates demonstrated co-resistance to at least two additional antimicrobial agents. Mkrtchyan et al. [7] cultured samples of *Staphylococcus spp.* obtained from toilets in four different public buildings. They reported that although these species may commonly be found in restrooms due to normal shedding of skin by washroom users, over a third of the isolates in their study carried multiple antibiotic resistance determinants. Over the past decade, strains of community-associated MRSA have been increasingly implicated in skin and soft tissue infections nationwide in the US and posed a significant public health challenge [4]. Whilst no MRSA was detected in this study, one methicillin resistant strain each of *S. epidermidis* (MRSE) and *S. saprophyticus* were isolated. The ability of bacteria to form ‘resistomes’ can facilitate persistent drug resistance in bacteria in the environment. Closely associated groups of bacteria can also share and maintain drug resistance determinants within suitable environments [7].

We observed that rubbish bins in the washrooms investigated were frequently uncovered and garbage was exposed to the washroom environment. Almost 20% of such bins were positioned immediately underneath warm air hand dryers and therefore at risk of dispersal of rubbish during operation of hand dryers. This potential aerosolization of contaminants from rubbish bins poses a health risk and should be avoided. Bacteria-contaminated air has been shown to be emitted whenever a warm-air dryer was running [24]. However, compared with warm-air dryers, air blade dryers have been reported to produce more ballistic droplets and have the potential to retain pathogenic bacteria on the hands and body of users [25]. Bacteria can also be distributed into the general environment and inhaled whenever dryers are operated [9]. Aerosol generation of contaminated air may be exacerbated if the rubbish bins without covers are placed in close proximity to the hand dryers.

The use of MALDI-TOF MS in our study facilitated the identification of pathogens in a matter of minutes instead of hours as would be required if using conventional methods. MALDI TOF can efficiently and accurately identify unknown species within a diversity of microbes; thus, it is suitable for environmental monitoring [26]. The hands can serve as vehicles for place-to-place and person-to-person propagation of microorganisms. Given the results from the environmental culturing, contamination of hands and other areas may occur in washrooms via contact with door handles, hand dryers and paper towel dispensers. Frequent cleaning of these facilities should be carried out. To minimise the possibility of cross-contamination among users and to decrease the opportunity to re-contaminate hands during and after handwashing, future washroom designs may consider using no-door washrooms, automatic controlled or hands-free paper towel dispensers or hands-free faucets with motion sensor. Other considerations for public washrooms include the provision of spare tissue rolls, toilet seat disinfectant, sufficient benches or shelves for placing belongings during handwashing and increasing the visibility of hand sanitizers and paper towels to washroom users.

Our study has some limitations. Firstly, as there were high numbers of bacterial colonies recovered from paper towel dispensers, hand dryers and door handles, we could not perform bacterial identification of every isolated colony. Instead, we randomly picked three colonies with distinctive morphology on TSA for identification. The proportion of bacterial species obtained in this study may not entirely reflect the species distribution in the sites. Secondly, due to resource limitation, the drug susceptibilities of gram negative rods were not determined. Thirdly, the data was collected via convenience sampling of fifty-five public washroom in Hong Kong. This may limit the generaliability of the findings.

The results from this study can be used to inform relevant stakeholders, including policymakers of ways to improve washroom layout to facilitate improved hygiene. Facility managers may wish to use the findings to reinforce the importance of frequent cleaning and maintenance of equipment by cleaning staff. There is a need for increased resources for low category washroom as these were signing less clean for each site tested. This study can also provide awareness to the general public and researchers of the overlooked areas of public washrooms.

## Conclusion

The findings of this study raise concerns about the planning and design as well as the cleanliness of public washrooms. Poor design and inappropriate positioning of washroom facilities may encourage bacterial contamination of the washroom environment and cause post-handwashing contamination of cleaned hands. Hand-drying facilities in public washrooms can act as reservoirs of drug-resistant bacteria. The importance of frequent cleaning and maintenance of public washrooms to promote safe hand hygiene practices for the public must be emphasised.

## Additional files

Additional file 1: **Table S1.** The general condition and facilities of the public washrooms (*n* = 55). (DOCX 17 kb)

Additional file 2: **Table S2.** Association between specific variables (category of washrooms, gender) and cleanliness^(1)^ of washroom facilities and environment (DOCX 54 kb)

Additional file 3: **Table S3.** Bacterial species isolated from washroom facilities (DOCX 30 kb)

## Abbreviations

CFU: Colony forming units
MALDI-TOF MS: Matrix-assisted laser desorption ionisation time-of-flight mass spectrometry
MRSA: Methicillin-resistant *Staphylococcus aureus*
SD: Standard deviation
SPP: Species
TSA: Tryptone Soy Agar
