# Supplementary material for: The public washroom - friend or foe? An observational study of washroom cleanliness combined with microbiological investigation of hand hygiene facilities
Source: Antimicrob Resist Infect Control. 2019 Feb 28;8:47. doi: 10.1186/s13756-019-0500-z (PMC6396476; doi:10.1186/s13756-019-0500-z)
Supplement: Supplementary file 1 — Table S1. The general condition and facilities of the public washrooms (n = 55). (DOCX 17 kb) [file 13756_2019_500_MOESM1_ESM.docx]

**Supplementary Table 1 The general condition and facilities of the public washrooms (*n*=55).**

| 1. **General condition** | **Number (%)** |
| --- | --- |
| **1.1 Washrooms of both genders (n=55)** | |
| Washroom category  High class  Middle class  Low class | 8 (14.5%)  36 (65.5%)  11 (20%) |
| Gender  Female toilet  Male toilet | 28 (51.0%)  27 (49.0%) |
| Door types  Automatic control  No door  With door, but open  With door, but close | 2 (3.6%)  10 (18.2%)  22 (40.0%)  21 (38.2%) |
| Number of toilet cubicles | Range: 2 to 11  Mode: 3 |
| Tissue rolls  Present  Absent | 55 (100%)  0 (0%) |
| Location of tissue rolls  Outside toilet cubicle  Inside toilet cubicle | 15 (27.3%)  40 (72.7%) |
| Spare tissue rolls  Present  Absent | 11 (20%)  44 (80%) |
| Toilet seat disinfectant  Present  Present but empty  Absent | 12 (21.8%)  2 (3.6%)  41 (74.6%) |
| Cleanliness of toilet (seat and drum)  Clean  Acceptable  Dirty | 20 (36.4%)  23 (41.8%)  12 (21.8%) |
| Floor condition  Clean  Acceptable  Dirty (with perceptible dirt and trash) | 19 (34.6%)  28 (50.9%)  8 (14.5%) |
| Mirror condition  Clean  Acceptable  Dirty (with obvious smudges or stains) | 23 (41.8%)  23 (41.8%)  9 (16.4%) |
| Walls condition  Clean  Acceptable  Dirty (with markings) | 25 (45.5%)  23 (41.8%)  7 (12.7%) |
| Sink condition  Clean  Acceptable  Dirty | 25 (45.5%)  22 (40.0%)  8 (14.5%) |
| Countertop condition  Clean  Reasonable  Dirty | 13 (23.6%)  31 (56.4%)  11 (20.0%) |
| Rubbish bin  Lid open  Lid closed | 49 (89.1%)  6 (10.9%) |
| Garbage location  Inside rubbish bin  Outside rubbish bin (e.g. on the floor, countertop) | 44 (80.0%)  11 (20.0%) |
| Location of rubbish bin  Right below hand dryer  Away from hand dryer | 10 (18.2%)  45 (81.8%) |
| Availability of shelf/area for placing personal items  Present  Absent | 25 (45.5%)   1. (54.5%) |
| Smell  Fresh/Fragrant  No special smell  Foul/Putrid | 29 (52.7%)  23 (41.8%)  3 (5.5%) |
| Cleaning log book  Present  Absent | 13 (23.6%)  42 (76.4%) |
| Overall cleanliness  (0 to 5, spotless to unacceptable) | Mean (SD):  1.55 (1.35)  Mode: 1 |
| Washroom temperature (^o^C) | Mean (SD):  27.51 (1.85) |
| Washroom humidity (%) | Mean (SD):  62.36 (10.76) |
| **1.2 Male washrooms only (n=27)** | |
| Number of urinals | Range: 2 to 11  Mode: 3 |
| Cleanliness of urinals  Clean  Acceptable  Dirty | 6 (22.2%)  15 (55.6%)  6 (22.2%) |
| Urinal cake  Present  Absent | 3 (11.1%)  24 (88.9%) |
| **1.3 Female washrooms only (n=28)** | |
| Sanitary bin  Lid open  Lid not properly closed  Lip completely closed | 13 (46.4%)  5 (17.9%)  10 (35.7%) |
| Sanitary disposal condition  Used napkins disposed inside bin  Used napkins overfill  Used napkins outside bin/on floor | 25 (89.3%)  2 (7.1%)  1 (3.6%) |
| 1. **Handwashing facilities** | **Number (%)** |
| Handwashing signage/reminder  Present  Absent | 8 (14.5%)  47 (85.5%) |
| Hand soap/detergent  Present  Absent | 53 (96.4%)  2 (3.6%) |
| Visibility of hand sanitizer dispenser to users  Yes  Hidden φ | 44 (80.0%)  11 (20.0%) |
| If the hand sanitizer dispenser was hidden, did the position for getting the hand sanitizer correctly indicated? (φ)  Yes  No | 3 (27.3%)   1. (72.7%) |
| Type of faucet  Hand faucet  Elbow control  Hands-free (with motion sensor) | 3 (5.5%)  3 (5.5%)  49 (89.1%) |
| 1. **Hand drying facilities** | **Number (%)** |
| Paper towels  Present  Absent | 40 (72.7%)  15 (27.3%) |
| Model of paper towels dispenser  Manual  Autonomic-controlled  Screw-controlled or lever-controlled | 32 (80%)  2 (5.0%)  6 (15.0%) |
| Visibility of paper towels dispenser to users  Yes  Hidden φ | 32 (58.2%)  23 (41.8%) |
| If the paper towels dispenser was hidden, did the position for getting the paper towels correctly indicated? φ  Yes  No | 13 (56.5%)  10 (43.5%) |
| Did the paper towels can be easily pulled out by users?  Yes  No | 40 (72.7%)  15 (27.3%) |
| Warm hand dryer  Present  Absent | 42 (76.4%)  13 (23.6%) |
| Model of warm hand dryer  Autonomic-controlled  Hand-controlled | 35 (83.3%)  7 (16.7%) |
| Jet hand dryer  Present  Absent | 6 (10.9%)  49 (89.1%) |
