# Supplementary material for: The public washroom - friend or foe? An observational study of washroom cleanliness combined with microbiological investigation of hand hygiene facilities
Source: Antimicrob Resist Infect Control. 2019 Feb 28;8:47. doi: 10.1186/s13756-019-0500-z (PMC6396476; doi:10.1186/s13756-019-0500-z)
Supplement: Supplementary file 2 — Table S2. Association between specific variables (category of washrooms, gender) and cleanliness(1) of washroom facilities and environment (DOCX 54 kb) [file 13756_2019_500_MOESM2_ESM.docx]

**Supplementary Table 2: Association between specific variables (category of washrooms, gender) and cleanliness^(1)^ of washroom facilities and environment**

| Cleanliness of: | Category | | | Clean | | Acceptable | | | Dirty | | Test statistics^#^  (Fisher’s Exact Test unless indicated)  *p* value^(2)^ |  |
| --- | --- | --- | --- | --- | --- | --- | --- | --- | --- | --- | --- | --- |
| Toilet seat and bowl | High | | | 7 | | 1 | | | 0 | | 0.001** |  |
|  | Middle | | | 13 | | 16 | | | 7 | |  |  |
|  | Low | | | 0 | | 6 | | | 5 | |  |  |
| Washroom floor | High | | | 6 | | 2 | | | 0 | | 0.000*** |  |
|  | Middle | | | 12 | | 22 | | | 2 | |  |  |
|  | Low | | | 1 | | 4 | | | 6 | |  |  |
| Walls | High | | | 8 | | 0 | | | 0 | | 0.001** |  |
|  | Middle | | | 16 | | 17 | | | 3 | |  |  |
|  | Low | | | 1 | | 6 | | | 4 | |  |  |
| Wash hand basins | High | | | 7 | | 1 | | | 0 | | 0.024* |  |
|  | Middle | | | 16 | | 16 | | | 2 | |  |  |
|  | Low | | | 2 | | 5 | | | 4 | |  |  |
| Urinals | High | | | 2 | | 2 | | | 0 | | 0.136 |  |
|  | Middle | | | 4 | | 11 | | | 2 | |  |  |
|  | Low | | | 0 | | 2 | | | 4 | |  |  |
|  |  | | |  | |  | | |  | |  |  |
|  |  | | | Fresh/  Fragrant | | No special smell | | | Foul/  Putrid odour | | 0.000*** |  |
| Smell/Odour in washroom | High | | | 8 | | 0 | | | 0 | |  |  |
|  | Middle | | | 20 | | 15 | | | 1 | |  |  |
|  | Low | | | 1 | | 8 | | | 2 | |  |  |
|  |  | | | Present | | Absent | | |  | |  | |
| Provision of toilet seat disinfectant | High | | | 3 | | 5 | | |  | | 0.067 |  |
|  | Middle | | | 11 | | 25 | | |  | |  |  |
|  | Low | | | 0 | | 11 | | |  | |  |  |
| Overall cleanliness  (0 to 5, spotless to unacceptable)  Mean (Standard deviation) | | High: 0.38 (0.74) | | | | | Kruskal-Wallis test:  X^2^ = 17.82, df=2, *p*<0.0001 *** | | | | |  |
|  |  | Middle: 1.36 (1.05) | | | | |  |  |  |  |  |  |
|  |  | Low: 3.00 (1.41) | | | | |  |  |  |  |  |  |
|  | |  |  | | |  |  | | |  | |  |
|  | |  | Clean | | Acceptable | | | Dirty | |  | |  |
| Cleanliness of toilet (seat and drum) | | Male | 5 | | 12 | | | 10 | | 0.006** | |  |
|  |  | Female | 15 | | 11 | | | 2 | |  |  |  |
| Floor condition | | Male | 11 | | 9 | | | 7 | | 0.014* | |  |
|  |  | Female | 8 | | 19 | | | 1 | |  |  |  |
| Wall | | Male | 14 | | 7 | | | 6 | | 0.026* | |  |
|  |  | Female | 11 | | 16 | | | 1 | |  |  |  |
|  | |  |  | |  | | |  | |  | |  |
| Countertop around wash hand basin | | Male | 5 | | 11 | | | 11 | |  | |  |
|  | | Female | 8 | | 20 | | | 0 | | 0.000*** | |  |

^#^only significant values were reported

Footnote^(1)^ : Cleanliness was determined by (a) absence of visible dirt and (b) splashes of water; ‘clean’ refers to the condition that is totally free of visible dirt and water; and ‘dirt’ refers to the specific region with any level of visible dirt and splashes of water; and ‘acceptable’ implies that condition between the two.

Footnote^(2)^: *, ** and *** refers to the level of significance in p value. * *p* ≤ 0.05, ** *p* ≤0.01, *** *p* ≤0.001.
