# Supplementary material for: The public washroom - friend or foe? An observational study of washroom cleanliness combined with microbiological investigation of hand hygiene facilities
Source: Antimicrob Resist Infect Control. 2019 Feb 28;8:47. doi: 10.1186/s13756-019-0500-z (PMC6396476; doi:10.1186/s13756-019-0500-z)
Supplement: Supplementary file 3 — Table S3. Bacterial species isolated from washroom facilities (DOCX 30 kb) [file 13756_2019_500_MOESM3_ESM.docx]

**Supplementary Table 3: Bacterial species isolated from washroom facilities.**

|  |  |  |  | No. of strains identified with the Biotyper^#^ | |
| --- | --- | --- | --- | --- | --- |
| Code | Bacterial identification  (alphabetical order) | Site | No. of isolates | Species level | Genus level only |
| 1 | *Acinetobacter lwoffii^(1)^* | c | 1 | 0 | 1 |
| 2 | *Aerococcus viridans* | c | 1 | 0 | 1 |
| 3 | *Bacillus altitudinis* | a, b, f | 3 | 1 | 2 |
| 4 | *Bacillus cereus* | b, c, d, f | 15 | 13 | 2 |
| 5 | *Bacillus circulans* | c | 1 | 0 | 1 |
| 6 | *Bacillus flexus* | c | 1 | 0 | 1 |
| 7 | *Bacillus infantis* | f | 1 | 1 | 0 |
| 8 | *Bacillus licheniformis* | c | 2 | 1 | 1 |
| 9 | *Bacillus marisflavi* | c | 1 | 1 | 0 |
| 10 | *Bacillus megaterium* | c | 1 | 1 | 0 |
| 11 | *Bacillus mycoides* | a | 1 | 0 | 1 |
| 12 | *Bacillus okuhidensis* | c | 1 | 1 | 0 |
| 13 | *Bacillus subtilis* | a, c, | 3 | 1 | 2 |
| 14 | *Brevibacterium casei* | a | 2 | 1 | 1 |
| 15 | *Corynebacterium falsenii* | c | 1 | 0 | 1 |
| 16 | *Enterobacter cloacae* | f | 1 | 1 | 0 |
| 17 | *Escherichia coli* | c, f | 3 | 2 | 1 |
| 18 | *Exiguobacterium aurantiacum* | c | 1 | 0 | 1 |
| 19 | *Kocuria kristinae* | a, f | 2 | 2 | 0 |
| 20 | *Kocuria palustris* | c, d, e | 3 | 1 | 2 |
| 21 | *Kocuria rhizophila* | b | 1 | 1 | 0 |
| 22 | *Micrococcus luteus* | a, b, c, d, f | 30 | 22 | 8 |
| 23 | *Moraxella sp* | c | 1 | 0 | 1 |
| 24 | *Moraxella_sg_Moraxella osloensis* | a, b, c | 6 | 1 | 5 |
| 25 | *Neisseria flavescens* | c, d, f | 3 | 2 | 1 |
| 26 | *Neisseria lactamica* | b | 1 | 0 | 1 |
| 27 | *Neisseria sp* | a | 1 | 0 | 1 |
| 28 | *Neisseria subflava* | c | 1 | 1 | 0 |
| 29 | *Pluralibacter gergoviae* | a | 1 | 0 | 1 |
| 31 | *Proteus mirabilis* | f | 1 | 1 | 0 |
| 32 | *Pseudomonas stutzeri* | c | 1 | 1 | 0 |
| 33 | *Roseomonas mucosa* | f | 1 | 1 | 0 |
| 34 | *Rothia aeria* | c | 2 | 1 | 1 |
| 35 | *Rothia amarae* | a | 1 | 0 | 1 |
| 36 | *Rothia dentocariosa* | b | 1 | 1 | 0 |
| 38 | *Staphylococcus aureus* | c, f | 3 | 2 | 1 |
| 39 | *Staphylococcus capitis* | b, d, f | 4 | 4 | 0 |
| 40 | *Staphylococcus cohnii* | c | 3 | 0 | 3 |
| 41 | *Staphylococcus condimenti* | f | 1 | 1 | 0 |
| 42 | *Staphylococcus epidermidis* | a, c, d, f | 22 | 12 | 10 |
| 43 | *Staphylococcus haemolyticus* | a, b, c, f | 13 | 9 | 4 |
| 44 | *Staphylococcus hominis* | a, b, c, d, f | 16 | 12 | 4 |
| 45 | *Staphylococcus kloosii* | C, f | 3 | 0 | 3 |
| 46 | *Staphylococcus lugdunensis* | a, e, f | 2 | 2 | 0 |
| 47 | *Staphylococcus pasteuri* | a, c, d, f | 14 | 8 | 6 |
| 49 | *Staphylococcus warneri* | a, c, e, f | 33 | 11 | 22 |
| 50 | *Staphyococcus saprophyticus* | c, d | 5 | 3 | 2 |
| 51 | *Streptococcus salivarius* | b | 1 | 0 | 1 |
| 52 | *Tsukamurella paurometabola* | e | 1 | 0 | 1 |
| **Total** | **52** |  | **218** | **123** | **95** |

Site: a = paper towel dispenser; b=paper towel; c=warm air dryer; d=jet air dryer (region around air outlet); e=jet air dryer (water receiver); f=internal door handle

^#^ Species level (high confidence identification possible, i.e. 2.00-3.00); Genus level (low confidence identification possible, i.e. 1.70-1.99)

^(1)^This is normal skin flora, except in immunocompromised catheter- related infection
